# Gastrointestinal Symptoms in Children With Life-Limiting Conditions Receiving Palliative Home Care

**DOI:** 10.3389/fped.2021.654531

**Published:** 2021-03-31

**Authors:** Holger Hauch, Peter Kriwy, Andreas Hahn, Reinhard Dettmeyer, Klaus-Peter Zimmer, Bernd Neubauer, Sabine Brill, Vera Vaillant, Jan de Laffolie, Kristine Schaefer, Irina Tretiakowa, Michaela Hach, Ulf Sibelius, Daniel Berthold

**Affiliations:** ^1^Palliative Care Team for Children, University Children's Hospital, Justus Liebig University Giessen, Giessen, Germany; ^2^Department for Pediatrics, Hospital of Bad Hersfeld, Bad Hersfeld, Germany; ^3^Institute for Sociology, Technical University of Chemnitz, Chemnitz, Germany; ^4^Pediatric Neurology, University Children's Hospital, Justus Liebig University Giessen, Giessen, Germany; ^5^Institute for Forensic Medicine, Justus Liebig University Giessen, Giessen, Germany; ^6^General Pediatrics and Neonatology, University Children's Hospital, Justus Liebig University Giessen, Giessen, Germany; ^7^Pediatric Oncology, University Children's Hospital, Justus Liebig University Giessen, Giessen, Germany; ^8^Association for Specialized Palliative Home Care, Wiesbaden, Germany; ^9^Internal Medicine IV/V, University Hospital, Justus Liebig University Giessen, Giessen, Germany

**Keywords:** home-based palliative care, vomiting, constipation, children, symptom burden

## Abstract

**Context:** Children with life-limiting diseases suffer from gastrointestinal (GI) symptoms. Since the introduction of specialized palliative home care (SPHC) in Germany, it is possible to care for these children at home. In phase 1 of care the aim is to stabilize the patient. In phase 2, terminal support is provided.

**Objectives:** Analysis were performed of the differences between these phases. The causes and modalities/outcome of treatment were evaluated.

**Methods:** A retrospective study was performed from 2014 to 2020. All home visits were analyzed with regard to the abovementioned symptoms, their causes, treatment and results.

**Results:** In total, 149 children were included (45.9% female, mean age 8.17 ± 7.67 years), and 126 patients were evaluated. GI symptoms were common in both phases. Vomiting was more common in phase 2 (59.3 vs. 27.1%; *p* < 0.001). After therapy, the proportion of asymptomatic children in phase 1 increased from 40.1 to 75.7%; (*p* < *0.001*). Constipation was present in 52.3% (phase 1) and 54.1% (phase 2). After treatment, the proportion of asymptomatic patients increased from 47.3 to 75.7% in phase 1 (*p* < 0.001), and grade 3 constipation was reduced from 33.9 to 15% in phase 2 (*p* < 0.05).

**Conclusion:** Painful GI symptoms occur in both palliative care phases but are more common in phase 2. The severity and frequency can usually be controlled at home. The study limitations were the retrospective design and small number of patients, but the study had a representative population, good data quality and a unique perspective on the reality of outpatient pediatric palliative care in Germany.

## Introduction

Children, adolescents and young adults with life-limiting diseases suffer from various symptoms at the end of their lives, which can be painful and impair their quality of life (1). Due to changes in the palliative care landscape in Germany, the conditions needed for the better recognition and treatment of these symptoms at home are emerging.

1. In Germany, progress has been made in the development of infrastructure that has led to improvements in the care of pediatric patients who need palliative care (2). In the outpatient sector, since 2007, patients with statutory health insurance in Germany have had a legal right to specialized palliative home care (SPHC) (3). In the ensuing years, many SPHC teams have been established for the care of children and young people (4). In contrast to SPHC for adults, which mainly treats older or very elderly patients with advanced cancer, SPHC teams for children and adolescents see a wide range of different and often rare diseases (2, 5). In SPHC for adults, there are usually rather shorter treatment periods, and patients die quickly (6). In pediatric palliative care, it is not uncommon to observe longer periods of care, which in 30–40% of cases lead to a stabilization of the patient's condition, after which the SPHC services can even be paused (5), leading to intermittent periods of care occur. In addition to crisis intervention, SPHC also has an element of care for the patients during their lives (= phase 1 of the SPHC “life care”).
2. This new care structure with the option of SPHC led to fewer affected children and adolescents dying in hospitals and more dying at home (1, 7). In England, between 1993 and 2014, the proportion of pediatric oncology patients who died in the hospital remained fairly constant at 50% (8). In another study in England, the proportion of children treated in a pediatric intensive care unit receiving terminal home care doubled to 33% when concurrent was provided by palliative care teams (9). In Germany, it was reported that children and young people who were cared for by an SPHC team died in their preferred place in 96% of cases (7). In adults, it was found that the proportion of terminally ill patients who died in the hospital decreased from 24.7 to 13.3% when SPHC support was available (10). Therefore, the SPHC teams are increasingly becoming involved in supporting patients in their home environments (= phase 2, “end-of-life care”).

In recent years, palliative care at home has played a more important role; therefore, it seems increasingly important that the burden of symptoms, their treatment and their outcomes are better recorded and investigated in the outpatient sector in both phase 1 and phase 2.

The symptoms include pain, restlessness, anxiety, fatigue, seizures and symptoms of the gastrointestinal tract (11, 12). In end-of-life care, children often exhibit many severe forms of these painful symptoms (1). Symptoms of the gastrointestinal tract can be treated with varying degrees of success in pediatric palliative care patients (11, 13). However, previous publications have mostly been limited to children with terminal cancer. Therefore, it is important to determine whether and to what extent the complaints of non-oncology patients can be treated in the outpatient setting and whether there are differences between the phases. In the authors' view, there is an urgent need to specifically investigate the quality of symptom control in SPHC and thus the improvement in patient quality of life (14, 15).

One particular aspect in the care of children with life-limiting illnesses is nutrition. Breastfeeding and feeding are essential elements in building parental relationships. A reduction in calories or food quantity that might alleviate symptoms could be perceived as a possible “beginning of the end” and therefore be rejected (16, 17). There are limited data on the diets of children treated in a palliative care setting. Nutritional problems (e.g., quantity, calorie content, type of feeding, actual energy intake requirements of the children) are highly individual in this very heterogeneous group of patients based on the medical context and the psychosocial environment. Swallowing problems, gastroesophageal reflux, feeding problems and refusal to eat can occur in combination with constipation, diarrhea and vomiting. These complaints can contribute to malnutrition with all its consequences (e.g., muscle weakness, susceptibility to infections, fatigue, and pressure sores) (18).

One particular patient with pronounced malnutrition and severe gastrointestinal symptoms provided the impetus for initiating this study. Due to the rarity of this patient's disease, the onset of the disease in early childhood, the availability of a pediatric care network (with a pediatrician as general practitioner and a link to special pediatric outpatient clinics) as well as the patient's child-like disposition, the pediatric body measurements and the family setting, this particular young adult was cared for by the reporting palliative care team for children and adolescents despite being older than 21 years.

### Case Description

The 28-year-old patient was observed at an early age to have delayed psychomotor development. The milestones of child development were not reached on time, and from school age onwards, developmental regression appeared in the form of childhood dementia. The girl lost her ability to longer walk, and her thinking and memory skills slowly but inexorably diminished over the years. At the same time, there were unpredictable phases during which she experienced difficulty swallowing and the sudden onset of abdominal pain and constipation, which made the patient miserable. Muscle biopsies and molecular genetic techniques were used to establish a diagnosis of mitochondrial neurogastrointestinal encephalopathy. Repeated aspiration events that caused severe pneumonia were observed. After repeated inpatient hospital treatments, a desire was expressed for home-based care. After an initial assessment and definition of objectives (e.g., securing home treatment, attempting to control symptoms, agreeing on emergency management), the patient was admitted to the SPHC service. During the first phase, episodes of pneumonia were treated with antibiotics, inhaled treatments and physiotherapy. Vomiting and sudden phases of paralytic ileus were treated with various laxative measures, antiemetics, embrocations, the application of aromatic oils and various phytotherapeutic teas. While participating in an integrative workshop, the patient suddenly aspirated a piece of bread roll, and an acute bolus event occurred that led to cardiovascular arrest. This was treated immediately and successfully with cardiopulmonary resuscitation (CPR) by the staff at the facility. The patient recovered after inpatient therapy without any additional neurological limitations. To prevent a recurrence of this event, the possibility of providing her nutrition entirely via the existing percutaneous endoscopic gastrostomy (PEG) and stopping oral nutrition was discussed but ultimately rejected by the family and the team. The patient appeared to experience life-fulfilling joy by eating. In the months that followed, a continuous decline in her mental abilities and progressive malnutrition despite additional medical nutrition supplied via her PEG were observed, which meant that the patient developed ileus with, it is assumed, bacterial migration through the enlarged bowel loops, resulting in death attributed to sepsis. The long care period of more than 2 years was characterized by some happier periods interrupted by repeated crises associated with a significantly reduced quality of life. The particularly distressing GI symptoms did not seem to have been especially well-controlled. The patient's caregivers described symptoms such as pain, restlessness, seizures or fever clearly and confidently and perceived them as being different from the gastrointestinal symptoms such as vomiting, constipation or malnutrition. This was likely because the patient had suffered repeatedly from transport disorders of the GI tract since infancy, and the parents seemed to have become accustomed to them. The authors questioned the attitude of their own team and wondered whether the GI symptoms did not require greater attention from the healthcare providers.

The questions addressed this clinical study are based on the assumption that the GI complex of symptoms described above differs between the life-care phase (life care, phase 1), which is characterized by an initial symptom burden, albeit one that should be controllable, and the terminal-care phase (terminal care, phase 2), which is characterized by end-of-life symptoms.

### Questions

The main questions were how often and with what severity GI symptoms occurred in phases 1 and 2, what were the causes of those GI symptoms and how and with what success they were treated. An additional question pertained to the characteristics of the nutritional situation of these seriously ill children and adolescents during the two treatment phases.

## Patients and Methods

A retrospective analysis was performed with the patients who were cared for by the reporting pediatric palliative care team in the period from 15.11.2014 to 15.05.2020. The inclusion criteria were the presence of a life-limiting disease and the prescription of and need for SPHC. Palliative diagnoses, age, sex and ACT group (19) were all documented. ACT group 1 includes life-threatening diseases for which curative therapies exist but for which treatment failure is possible (examples: cancer or heart disease with curative options). Group 2 includes diseases involving longer periods of intensive treatment that are aimed at prolonging life and enabling participation in normal childhood activities but due to which premature death is likely (examples: cystic fibrosis, Duchenne muscular dystrophy). Group 3 includes progressive diseases with no clear therapeutic options for which palliative treatment is used (examples: certain forms of neuronal ceroid lipofuscinosis, mucopolysaccharidosis). Group 4 includes diseases that cause severe neurological disabilities; patients often suffer from complications of the underlying disease and deterioration can be unpredictably but not usually progressively (examples: perinatal asphyxia, congenital brain malformations).

All home visits and reassessments were evaluated with regard to gastrointestinal symptoms/nutrition (parameters: “vomiting,” “constipation,” “diarrhea,” “ascites”), the presence of a gastric stoma with a gastric and/or jejunal probe (PEG/PEJ), the presence of a central venous catheter (CVC), and the administration of medical (par)enteral nutrition. In cases of the clinical suspicion of symptomatic ascites, ultrasound was performed (Vscan®, GE Vingmed Ultrasound, N-3191 Horten, Norway). A record was kept of whether nutrition/liquid administration was stopped before death. If a complete data set was not available for the home visits, the recorded data were fed into the analysis, and the missing values were documented.

Data were entered sequentially and double-checked (two pediatric specialists). The severity of GI symptoms was assessed using a Likert scale (min. 1 = no symptom burden to max. 5 points) during the external assessment by the SPHC team. The classification of the severity of symptoms was based on the “Common Terminology Criteria for Adverse Events (CTCAE) Version 5.0” (20). The symptom burden at the beginning of phases 1 and 2, the maximum severity and the change in severity after treatment were documented.

The retrospective classification of the causes of the GI symptoms was also carried out using the same method with the following parameters: “underlying disease,” “infection,” “adverse effect,” “combination of different causes” and “unclear.” Malnutrition was diagnosed if the measured weight was > 3 standard deviations below the mean value of the reference population (21) or between the 2nd and 3rd standard deviations with an undesired weight loss > 5% of the starting weight (22).

Due to the large number of treatments used in the patients, therapies were evaluated in clusters (parameters: “medicinal measures,” “nursing measures,” “phytotherapeutic/complementary procedures,” “combination treatment,” “sedation of the patient” and “other measures”) (see Table 1).

**Table 1:** Treatment summary.

**Treatment**	**Vomiting**	**Constipation**	**Diarrhea**	**Ascites**
Drugs	Ondansetron Granisteron Scopalamine Dexamethasone	Lactulose Macrogol Bisacodyl Sodium picosulphate Erythromycin (in 1 case)	*Lactobacillus* spp. Loperamide Racecacodril	Spironlactone Furosemide
Nursing	Modification of nutrition Odor management Oral care	Adaptation of fluid intake Enema (sorbitol solution) Regular embrocations (according to Wegmann/Hauschka) (23)	Adaptation of fluid intake Odor management Oral care	Gentle positioning of the patient Odor management
Phytotherapy	Ginger tea	Warm and humid compresses with camomile, fennel oil, tea with fennel, caraway, anise	Compresses with peppermint oil	Compresses with peppermint oil
Sedation	Not done	Midazolam	Not done	Not done
Other treatment	Smaller food portions Nux vomica D6	High fiber nutrition	Adaptation of food proportions	Higher protein nutrition Ultrasound-guided puncture

**Figure 1:**
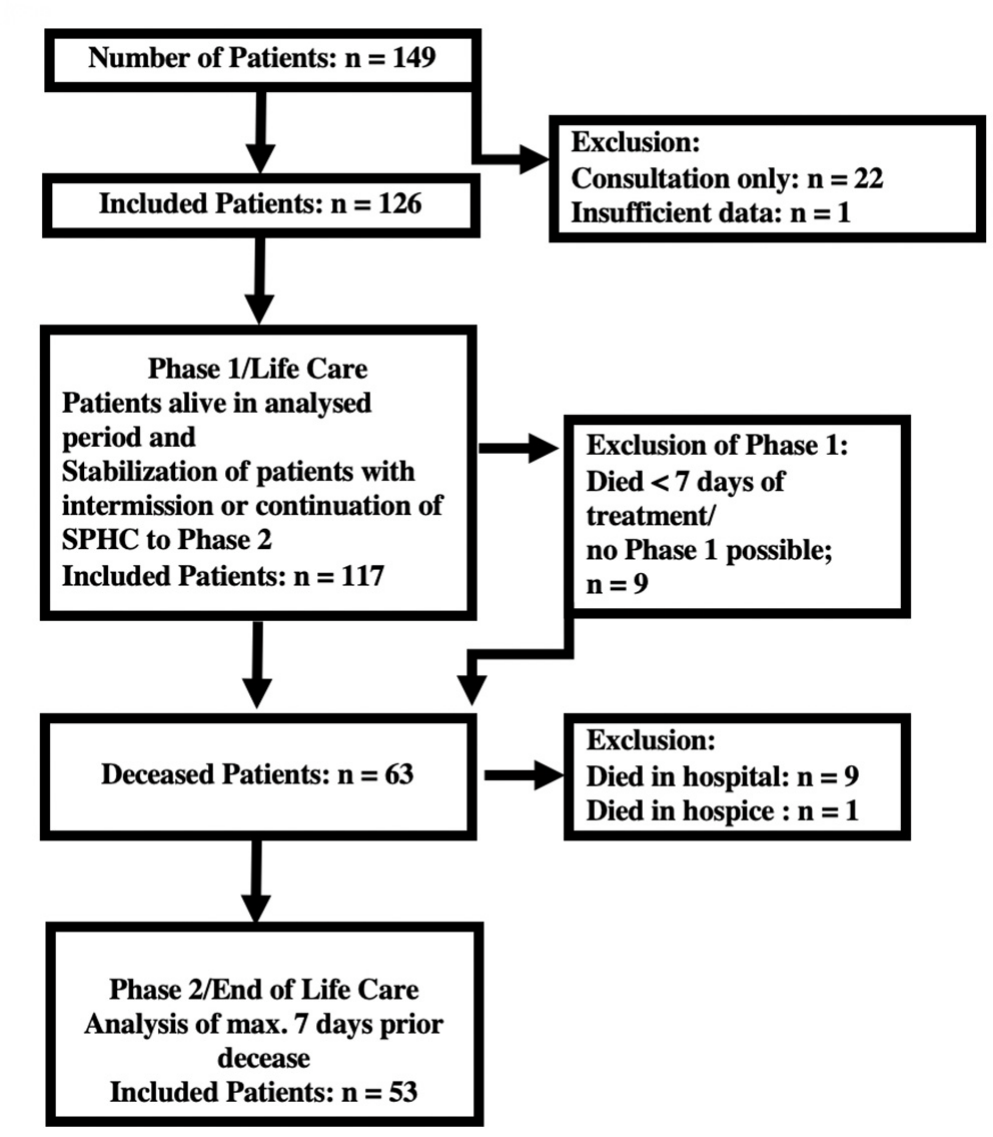
Inclusion of patients.

Assuming that the symptoms differ in phase 1 and phase 2, a classification system was established (see Figure 1). The data collected during the last 7 days of care were designated the terminal section of phase 2 (24). All other care sections were recorded and evaluated as belonging to phase 1. The exclusion criteria for phase 1 were exclusively terminal care and a duration of care <7 days; the exclusion criteria for phase 2 were death not occurring at home, hospital or SPHC environment (hospital, hospice) or family refusal to participate.

To check the representativeness of the study population, a comparison was made with anonymous data from all children and adolescents who were cared for in the SPHC system in the period from 2014 to 2018 across the entire federal state of Hesse (previously unpublished data, courtesy of the “Fachverband SAPV Hessen e.V.”).

From 01.11.2014 to 31.12.2015, the medical records were managed in the “KIS” hospital documentation system [KAOS, Justus Liebig University (JLU), Giessen, Germany], and from 01.01.2016 onwards, they were managed with “Pallidoc®” software (Stat-Consult, D-39104 Magdeburg, Germany). The analysis was carried out with anonymized data. The data were processed using SPSS 25.0 software (IBM Incorporation Armonk, NY 10504, United States) and statistically analyzed. Chi-square tests, non-parametric tests (Wilcoxon) and Spearman correlation analyses were carried out. Testing for significant differences between phases 1 and 2 was performed taking into account the dependence of the variables.

The legal guardians or the patients agreed to the collection of the data and its scientific evaluation. The ethics committee of JLU's Department 11 agreed to the performance of this study (ref. 78/20). This study was certified by the German Register for Clinical Studies (ID: DRKS00022332; https://www.drks.de/drks_web/).

## Results

### Patient Distribution/ACT Groups

In the study period, the pediatric palliative care team treated 149 patients. Twenty-two children and adolescents had to be excluded from the analysis of the home visits because only individual counseling appointments were made and no data were available about the disease course, therapy and treatment results. One child was only cared for in the terminal phase for a total of 4 h and was not analyzed (see Figure 1). The remaining 126 children were initially included in the study; 117 children were grouped together in phase 1. Nine children died within 7 days of admission to the SPHC services without stabilization of their condition. A total of 63 of the patients died, of whom 49 were receiving SPHC at home and 4 were in nursing homes. The data from the 9 children who died in the hospital and 1 who died in an inpatient hospice could not be evaluated (Figure 1), partly because the aim of the study was to analyze outpatient care.

In the analysis of the representativeness of the study population in comparison with a large similar population (*n* = 778) of all children and adolescents treated in the SPHC program in the federal state of Hesse (population approximately 6.3 million) from 2014 to 2018, no significant differences were found in terms of sex, age or ACT groups (Table 2).

**Table 2:** Patients.

	**Patients**	**Control group hesse 2014–2018**	**Life care (Stage 1)**	**End of life care (Stage 2)**
N	149	778	117	53
Sex	46.8 ♂/53.2 ♀	42.1 ♂/57.9 ♀	47.6 ♂/52.4 ♀	44.1 ♂/55.9 ♀
Age [years]	Ø 8.1 ± 7.6 Median: 5.21	Ø 8.3 ± 7.4 Median: 6.57	Ø 8.05 ± 7.67 Median: 5.1	Ø 8.48 ± 7.80 Median: 6.2
ACT groups [%]	1: 26.4 2: 8.0 3: 37.0 4: 28.6	1: 22.8 2: 10.0 3: 39.3 4: 27.9	1: **28.8** 2: 8.0 3: 36.0 4: 27.2	1: **42.9 (*****p<*** **0.05)** 2: 7.9 3: 31.7 4: 17.5

### Differences Between Phase 1 and Phase 2

The children in phase 2 had a significantly higher proportion in ACT group 1 (mainly malignant diseases) (42.9%) than the children in phase 1 (28.8%) (phase 1) [χ^2^ (6) = 14.494; *p* < 0.05].

Within the parameter of a defined maximum phase 2 duration of 7 days, the duration of phase 1 showed considerable variability (phase 1: mean value 162.5 +/– 277.1 days, median 59 days; phase 2: mean value 6.31 +/– 1.7 days, median 7 days). Over the study period, 4,028 planned home visits (HV), 591 home visits for crisis intervention (HV-C), 10,008 telephone visits (TV), and 2,391 telephone calls for crisis intervention (TV-C) were carried out in phase 1. In phase 2, 370 planned HV, 137 HV-C, 472 TV and 176 TV-C were carried out. Considering the significantly shorter duration of phase 2, significant differences can be observed in the number of contacts/week. In phase 2, there was a significant increase in the need for care. In phase 1, there were an average of 1.7 +/– 1.2/week HV (phase 2: 2.9 +/−1.7/week; *p* < 0.001), 0.5 +/– 1.2/week HV-C (phase 2: 3.7 +/– 8.2/week; *p* < 0.001), 3.7 +/– 3.1/week TV (phase 2: 5.3 +/– 4.6/week; *p* < 0.001) and 1.4 +/– 1.8/week TV-C (phase 2: 2.3 +/– 2.6/week; *p* < 0.001).

With regard to the parameters selected, the HV in both phases were fully documented in terms of the specific symptoms in phase 1 (96.9–97.7%) and phase 2 (96.1–98.3%).

GI symptoms were observed in both phases (see **Figure 3**). In general, the GI symptoms were of varying severity according to the CTCAE scale; grades 1–4 were recorded, but no cases of grade 5 GI symptoms (i.e., causative of the child's death).

### Vomiting

**Figure 2:**
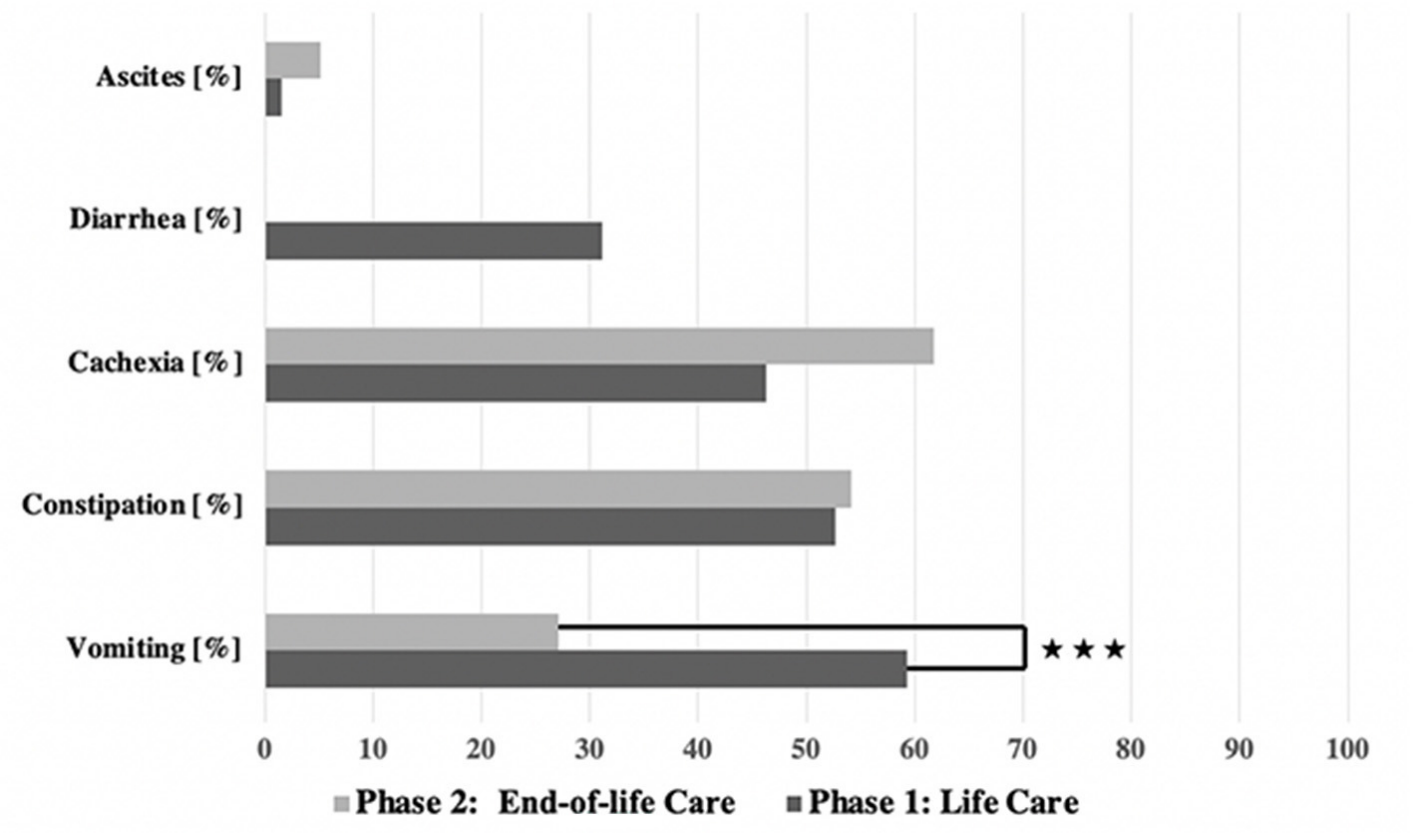
Frequency of symptoms in both groups. ***p < 0.001.

**Figure 3:**
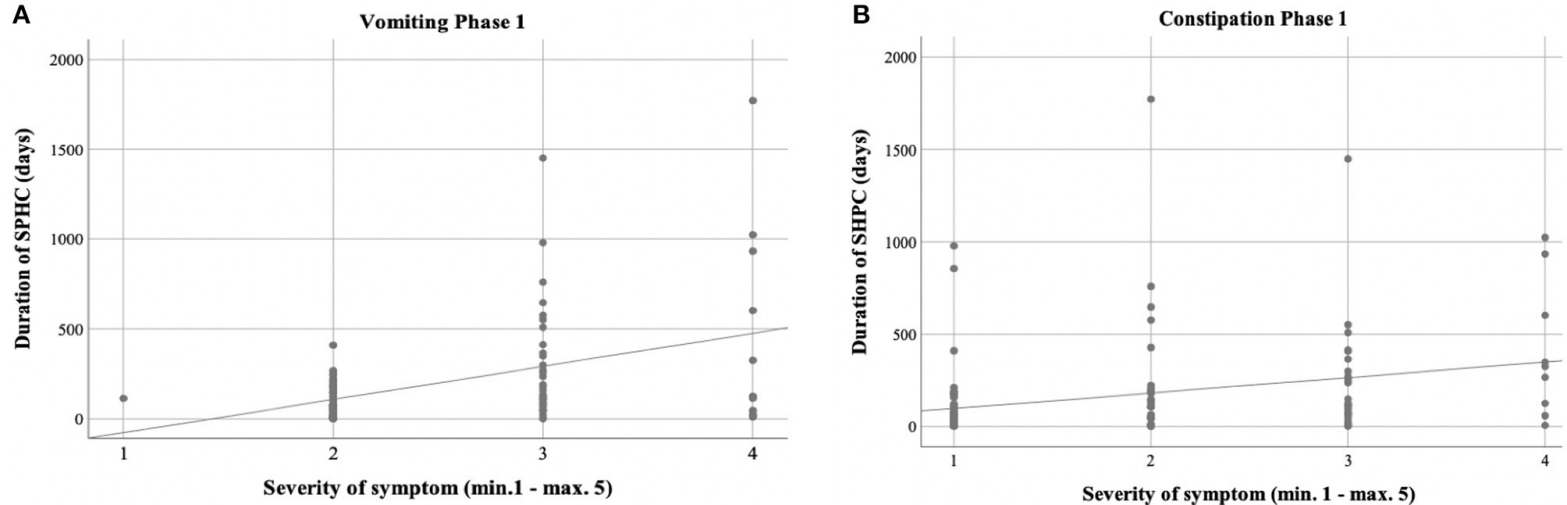
Correlation between duration of care and severity of symptoms. **(A,B)** Phase 1 showed positive correlation between the duration of sickness/care vomiting **(A)** (|r| = 0.346; *p* = 0.003; Spearman) and constipation **(B)** (|r| = 0.388; *p* = 0.001; Spearman).

**Figure 4:**
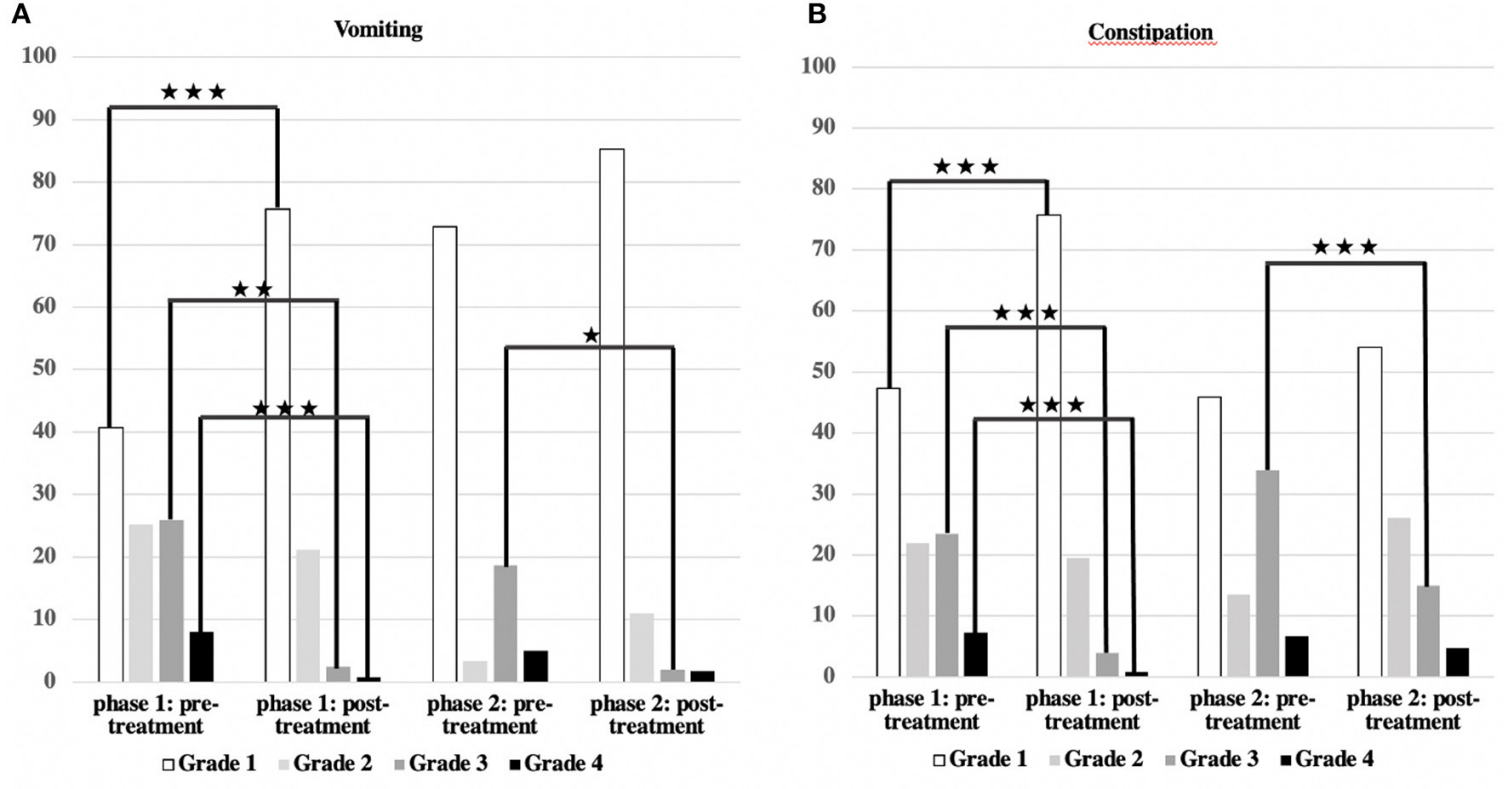
Severity of vomiting and constipation/effect of treatment. **(A,B)** The main symptoms, vomiting, and constipation, could be significantly reduced in both groups (***p < 0.001; **p < 0.01; *p < 0.05).

Vomiting occurred more frequently in phase 1 (59.3%) than in phase 2 (27.1%) [χ^2^ (2) = 17.244; *p* < 0.001] (Table 3, Figure 2). There was a correlation between the duration of sickness/care and the severity of vomiting in phase 1. The longer the treatment, the more frequently severe vomiting occurred (|r | = 0.346; *p* = 0.003; Spearman; see Figure 3A). The evaluation of the causes of vomiting showed no significant differences between phase 1 (underlying disease 54.8%; infection 15%; adverse effect 9.6%; combination 9.6%; unclear 11%) and phase 2 (underlying disease 75%; infection 6.4%; adverse effect 6.4%; combination 12.2%; unclear 0%) (*p* = 0.18). In phase 1, palliative therapy increased the proportion of asymptomatic patients (= grade 1) from 40.7 to 75.7% [χ^2^ (2) = 25.742; *p* < 0.001], reduced the proportion of patients with grade 4 vomiting from 8.1 to 0.8% [χ^2^ (2) = 30.437; *p* < 0.001] and reduced the proportion of patients with grade 3 vomiting from 26 to 2.4% [χ^2^ (2) = 8.823; *p* < 0.05]. In phase 2, the proportion of patients with grade 3 vomiting fell from 18.7 to 2% [χ^2^ (2) = 10.359 *p* < 0.01] (Figure 4A). The type of treatment did not differ significantly between phase 1 (antiemetic drug 60%, care/phytotherapeutic measures 6.7%, combination 33%) and phase 2 (antiemetic drug 83.4%, combination 16.6%).

**Table 3:** Causes/treatment of symptom vomiting.

	**Life Care (Stage 1)**	**End of Life Care (Phase 2)**	**Significance**
N	117	53	
Vomiting [%]	59.3	27.1	***p<*** **0.001**
Causes			
Underlying disease	54.8	75.0	n.s.
Infection	15.0	6.4	n.s.
Side effect	9.6	6.4	n.s.
Combination	9.6	12.2	n.s.
Unknown	11.0	0	n.s.
Treatment			
N/Missing values	126/3	63/3	
Drugs (D)	83.4	60.0	n.s.
Nursing (N)/Phytotherapy (P) alone	0	6.7	n.s.
Combination (D+/–N+/–P)	16.6	33.3	n.s.

p < 0.001 means that the difference is significant “yes, p < 0.001” (n.s. = not significant).

### Constipation

The frequency of constipation did not differ significantly between the two phases (52.7% in phase 1 and 54.1% in phase 2). In phase 2, adverse effects (mainly from opiate-based painkillers) occurred more frequently (42.4%) than in phase 1 [23.1%; χ^2^ (2) = 6.068, *p* < 0.05] (see Table 4). The effect was more pronounced in ACT group 1 than in ACT groups 2–4, with a higher rate of opiate-induced constipation [82.4% (ACT-1) vs. 23.5% (ACT 2–4)] [χ^2^ (2) = 10,806; *p* < 0.01]. Similar to vomiting, the duration of the disease correlated with the severity of constipation [|r| = 0.388; *p* < 0.001; Spearman, see Figure 3B]. In both phases, the therapy was predominantly a drug-based laxative (phase 1: 50.8%; phase 2: 54.5%). A combination of nursing, phytotherapeutic and pharmacological procedures was used in 49.2% (phase 1) and 42.5% (phase 2) of cases. In phase 1, after treatment, the proportion of asymptomatic patients increased from 47.3 to 75.7%, and the frequency of grade 3/grade 4 constipation was reduced from 23.5/7.3 to 4.0/0.8% [χ^2^ (4) = 20,532; *p* < 0.001]. In phase 2, after treatment, the proportion of patients with grade 3 constipation was reduced from 33.9 to 15.0% [χ^2^ (4) = 25.740 *p* < 0.001] (see Figure 4B).

**Table 4:** Causes/treatment of constipation.

	**Life Care (Stage 1)**	**End of Life Care (Phase 2)**	**Significance**
N	117	53	
Constipation [%]	52.3	54.1	n.s.
Causes			
Underlying disease	55.3	42.4	n.s.
Infection	0	3.0	n.s.
Side effect	23.1	42.4	*p <* 0.05
Combination	15.4	9.2	n.s.
Unknown	6.2	3.0	n.s.
Treatment			
Drugs	50.8	54.5	n.s.
Nursing (N)/Phytotherapy (P) alone	0	3.0	n.s.
Combination (D+/–N+/–P)	49.2	42.5	n.s.
Sedation	0	3.0	n.s.

### Other GI Symptoms

#### Diarrhea

Diarrhea was observed in 31.1% of cases only in phase 1. The cause was usually an infection (82.5%) (underlying illness 10%, adverse effect 7.5%). Level 4 diarrhea was found in 8.5% of the cases (grade 2: 13.3%; grade 3: 9.3%). In 74.4% of the cases, there was increased fluid intake, and in 25.6%, a combination of nursing measures and phytotherapeutic procedures was administered. Grade 4 diarrhea no longer occurred after treatment or after sufficient time had passed [8.5–0%, χ^2^ (2) = 10.637; *p* < 0.01].

#### Ascites

Ascites were observed in 1.6% (phase 1) and 5.1% (phase 2) (*p* = 0.067) of patients, exclusively in the ACT 1 group (oncology and cardiology patients) as a consequence of the underlying disease. A puncture was not necessary for any patient. The symptom burden caused by the ascites (feeling of pressure and shortness of breath) was stabilized but not improved by the use of pharmaceuticals (e.g., diuretics) or other procedures (see Table 1).

#### Type of Nutrition/Malnutrition

A PEG/PEJ system was present in 54.5% (phase 1) and 41.9% (phase 2) of the patients but was only used for nutrition in some patients (45.9% in phase 1; 28.6% in phase 2). In phase 1, 29.3% of the patients had a CVC, and 12.8% were fed parenterally. This proportion increased non-significantly in phase 2–38.7% of patients with a CVC and 15.9% receiving parenteral nutrition. Nearly all patients with a CVC had an underlying oncological or cardiological disease.

Malnutrition/cachexia tended to occur more frequently in phase 2 (phase 1: 46.3%; phase 2: 61.7%) [χ^2^ (2) = 4.235; *p* = 0.15]. With regard to cachexia, there were no differences between patients in ACT group 1 and those in groups 2–4 [ACT-1 53.6% vs. ACT 2/3/4 61.6%; χ^2^ (2) = 0.791; *p* = 0.7].

In one patient, nutrition and fluid administration was actively ceased by the family without the involvement of the pediatric palliative care team. This was a 14-year-old male adolescent with a severe brain malformation (lissencephaly) who, according to his parents, had shown a marked deterioration in his general condition over a period of weeks, with severe vomiting, fever and shortness of breath. The boy had vomited during any feeding and/or fluid intake, so the parents actively stopped feeding and fluid administration 4 days before his death. The pediatric palliative care team was only involved at a later stage, so that the first SPHC care session began only 4 h before the boy's death. During palliative medical care, no vomiting occurred, dyspnea was treated with opiate therapy, and the boy died with his symptoms controlled. Due to the brevity of the involvement of the SPHC team, the boy was not included in the database (see Figure 1, insufficient data).

## Discussion

Children and adolescents who were treated in the SPHC program often suffered from painful symptoms affecting the GI tract. There were differences between the stages. In phase 2 in the present cohort, there were more patients in the ACT 1 group, as the oncology patients in this group were usually only assigned to the SPHC program after all resources had been exhausted and they were at an advanced stage of the disease. The terminal phase was characterized by an increased need for support, as evidenced by the significantly increased number of TV and HV. The presumed differences in the distribution and severity of symptoms were observed in the previously defined phases. When comparing the two phases, it is important to bear in mind the considerable difference in the duration of the treatment phases (phase 1 median 59 days vs. phase 2 median 7 days), so that even if a symptom is equally frequent and pronounced, this is likely to place a greater burden on patients in phase 2 than in a longer phase in which it would be possible to achieve stabilization between episodes. Vomiting was more frequent in phase 1 and could be controlled, making this unpleasant symptom less common in phase 2.

Children and adolescents in the SPHC program are characterized by a high potential for crisis. In both phases, there are emergency HV and TC-C. For vomiting and constipation, it was found that the duration of the disease correlated with the severity of symptoms and could be controlled more successfully in the longer phase 1 than in the shorter phase 2. A correlation between symptoms and duration could not be shown simply because of the short duration of phase 2.

To date, the rate of GI symptoms in non-oncology patients has been rarely studied (25). Zernikow et al. reported the occurrence of this symptom in 40–63% (1) of patients. In an interview study of parents of deceased pediatric oncology patients, a rate of GI symptoms of 31% was reported (26). In the 1990s, moderate therapeutic success rates, e.g., for constipation or vomiting, were reported among oncology patients. Here, 7 distressing symptoms were seen in 92 children with cancer who later died in the terminal phase; 4 of those symptoms were related to the GI tract (appetite loss 36%, constipation 34%, nausea and vomiting 27% and diarrhea 22%). Constipation was successfully treated in only 10% of the patients (11).

In the analysis of vomiting, similar data with rates of 40–60% were found in different studies involving pediatric oncology patients (1, 11, 27, 28). Successful treatment of vomiting was reported in 10% of cases (11). In the group of SPHC patients examined in this study, 88.9 or 60% (phase 1/phase 2) of 43 patients with cancer were able to achieve control of their vomiting (severity grade 1 to max. grade 2).

In children with malignant disease, constipation was adequately treated; in 62.5%/47.1% (phase 1/phase 2), an improvement was achieved in constipation (maximum grade 2), and in 12.5%/5.9% (phase 1/phase 2), stool frequency could be normalized.

Compared to historical data, symptom control in children and adolescents with cancer appears to be more successful in the context of SPHC. Among the limitations of the retrospective study design, the close monitoring of progression and therapy by the SPHC with several telephone contacts per week and at least one multidisciplinary HV per week as well as the interdisciplinary and multiprofessional therapeutic approach involving nursing and phytotherapeutic/complementary elements may have contributed to this successful symptom control (see Table 1).

The reported incidence of diarrhea in the literature was 21–40% (1, 11, 27, 28), and the 31% found in this study is thus in the reported range. The symptoms occurred exclusively in phase 1 in the patients examined here. The reason for this could be the short duration of phase 2, which was by definition a maximum of 7 days, compared to the duration of phase 1. Diarrhea was considered to be of mostly infectious etiology, associated with antibiotic therapy or paradoxical diarrhea in patients with constipation and could therefore also have occurred in phase 2.

As expected, ascites occurred rarely and only in oncology or pediatric cardiology patients as an result of the progressive or complicated underlying disease, and it could therefore only be stabilized.

The evaluation of nutritional disorders or cachexia is only possible to a limited extent on the basis of the data available and the study design. The severity of cachexia in childhood has not yet been systematically described and classified (29). The study of cachexia, as a complex metabolic syndrome involving weight loss, lack of growth or loss of appetite (25, 30, 31) due to a decreasing feeling of hunger, is especially difficult in a dying process that may begin slowly (32). Malnutrition during palliative care did not increase significantly from phase 1 to phase 2, with 40% of children receiving medical feeding at the end of their lives. In the future, a prospective study would be helpful to examine the links between weight progression, calorie intake and symptoms such as (hyper)secretion, dyspnea, restlessness and GI symptoms. The perspective of the parents, who could also understand cachexia as a failure of their role as providers of food for their child, should be included in the consideration of the problem.

### Limitations of the Study

The first limitation is the small size of the group, with only 149 children studied. However, no differences in age, sex distribution or distribution of the ACT groups could be found between the study population and all of the children and adolescents cared for in the state of Hesse in the SPHC program from 2014 to 2018. A further comparison with data from the Düsseldorf working group showed a very similar distribution of ACT groups (12). The diseases leading to the need for pediatric SPHC are usually very rare, so the patient numbers are naturally much lower than those in the adult population, in which mostly older patients with common cancers are being treated. It can be assumed that the patients included in the analysis are representative of the group of children and adolescents with life-limiting illnesses who require SPHC in Germany.

The retrospective design is a limitation, as the documentation system does not contain standardized parameters for nutritional quantity and complementary treatments. Non-drug treatments and nutrition were freely documented, but a distorted, investigator-dependent reduced likelihood of the inclusion of these data can be assumed. However, all drug prescriptions and dose adjustments were recorded in the documentation system.

In the event of communication problems due to a language barrier, HV were occasionally accompanied by interpreters, but this can be a further limitation due to the potential for missing information. A total of 16.9% of the affected families had significant communication problems (ethnic origin: Syria 7.4%, Iran 2.0%, Eritrea/Somalia 2.0%, Balkan states 2.7%, Afghanistan 1.4%, Algeria 1.4%). Even among families who have lived in Germany for a longer period of time, regular HV were associated with language restrictions in 17.6% (Turkey 9.5%, Russia/former CIS: 5.4%, Italy: 2.7%). The retrospective classification of symptom severity according to the CTCAE scale does not appear to be meaningful, as grade 5 was not determined at any time, and the patients were not exclusively suffering from underlying oncological diseases. A retrospective change to more suitable scales, such as the Palliative Care Outcome Scale IPOS (33), could lead to a systematic distortion of the results. The recording of symptoms was switched to the IPOS at the end of the data collection of this presented work so that future examinations can be recorded with this instrument.

In summary, it can be concluded from these retrospective data that among the GI symptoms, constipation and vomiting in particular are of the greatest importance in the care of children and adolescents with life-limiting illnesses in the home environment. In this study, non-oncology patients suffering from no less severe GI symptoms than the oncology patients were also examined. In SPHC, there are two phases of care that have different characteristics with regard to symptoms of the GI tract. It is important to identify the various causes and to achieve rapid symptom relief. In the setting of SPHC, therapeutic success was adequate in the patients examined. Whether differences in other symptoms can also be found should be assessed in further analyses. Overall, there is a lack of prospective studies on the treatment of distressing symptoms in children with life-limiting diseases (34).

## Data Availability Statement

The raw data supporting the conclusions of this article will be made available by the authors, without undue reservation.

## Ethics Statement

The studies involving human participants were reviewed and approved by Ethikkommission des Fachbereichs Medizin Justus Liebig Universität Giessen. Written informed consent to participate in this study was provided by the participants' legal guardian/next of kin.

## Author Contributions

HH reviewed the literature, collected the data, performed the statistical analysis, contributed to ethics commission proposal, and drafted the manuscript. PK controlled the data, contributed to the statistical analysis, and reviewed the manuscript. AH, RD, K-PZ, BN, SB, VV, JL, KS, MH, IT, and US reviewed the literature, discussed the data, and contributed to the drafting the manuscript. DB controlled the data and contributed to the statistical analysis, reviewed the manuscript, and discussed the study design. All authors issued final approval for the version to be submitted.

## Conflict of Interest

The authors declare that the research was conducted in the absence of any commercial or financial relationships that could be construed as a potential conflict of interest.
